# Cross-species analysis of differential transcript usage in humans and chickens with fatty liver disease

**DOI:** 10.14202/vetworld.2023.1964-1973

**Published:** 2023-09-23

**Authors:** Kaj Chokeshaiusaha, Thanida Sananmuang, Denis Puthier, Catherine Nguyen

**Affiliations:** 1 Department of Veterinary Science, Faculty of Veterinary Medicine, Rajamangala University of Technology Tawan-OK, Chonburi, Thailand; 2 Aix-Marseille Université, INSERM, UMR 1090, TAGC, Marseille, France

**Keywords:** cross-species biomarkers, differential gene expression, differential transcript usage, fat metabolism, fatty liver disease, lipidosis, transcript isoforms

## Abstract

**Background and Aim:**

Fatty liver disease is a common condition, characterized by excess fat accumulation in the liver. It can contribute to more severe liver-related health issues, making it a critical concern in avian and human medicine. Apart from modifying the gene expression of liver cells, the disease also alters the expression of specific transcript isoforms, which might serve as new biological markers for both species. This study aimed to identify cross-species genes displaying differential expressions in their transcript isoforms in humans and chickens with fatty liver disease.

**Materials and Methods:**

We performed differential gene expression and differential transcript usage (DTU) analyses on messenger RNA datasets from the livers of both chickens and humans with fatty liver disease. Using appropriate cross-species gene identification methods, we reviewed the acquired candidate genes and their transcript isoforms to determine their potential role in fatty liver disease’s pathogenesis.

**Results:**

We identified seven genes - *ALG5*, *BRD7*, *DIABLO*, *RSU1*, *SFXN5*, *STIMATE*, *TJP3*, and *VDAC2* - and their corresponding transcript isoforms as potential candidates (false discovery rate ≤0.05). Our findings showed that these genes most likely contribute to fatty disease development and progression.

**Conclusion:**

This study successfully identified novel human-chicken DTU genes in fatty liver disease. Further research is encouraged to verify the functions and regulations of these transcript isoforms as potential diagnostic markers for fatty liver disease in humans and chickens.

## Introduction

Fatty liver disease is a common lipid metabolism disorder in chickens and humans characterized by excessive fat deposits in the liver. The fatty metamorphosis in chickens is comparable to nonalcoholic fatty liver disease (NAFLD) in humans [1]. Nonalcoholic fatty liver disease is a prevalent metabolic disorder in humans caused by excessive lipid accumulation in the liver [2]. Similar to NAFLD, chickens with fatty liver disease exhibit comparable physiological and pathological traits, marked by a disruption in lipid homeostasis processes, including hepatic lipid accumulation, transportation, and metabolism. Given these similarities, chickens with fatty liver disease are robust animal models for exploring NAFLD [1, 3]. Although generally considered benign, fatty liver disease can contribute to liver-related morbidity and mortality, making it a critical issue in avian and human medicine [1].

Recent transcriptomic studies have unraveled complex lipid mechanisms in humans [4–7] and chickens [8–10]. Differential gene expression (DGE) profiling of fatty liver samples from humans and chickens revealed multiple biological systems and pathways involved in the progression of fatty liver disease. Several orthologous genes shared between humans and chickens, namely *ACACA*, *APOA4*, *DPP4*, *FADS2*, *FASN*, and *SCD*, are involved in lipid accumulation, transportation, and metabolism, contribute to the pathogenesis of this disease in both species [6, 8–11]. Moreover, researchers have also found numerous differentially expressed genes involved in various cellular perturbations that indirectly impact the liver’s fat regulation [5, 6, 9]. This evidence highlights the complexity of the disease’s development, emphasizing the need for further investigations into the mechanisms underlying this disease.

Apart from direct modifications in total gene expression level, multiple transcript genes can show functional loss or aberrations due to isoform switching [12]. Transcript isoforms can have several roles, even opposing ones, which might be conserved or exclusive to the species. Hence, these isoforms are promising biomarkers for predicting disease prognosis and severity across different disorders since changes in their expression levels can critically contribute to the disease’s development [13, 14]. While several RNA splicing studies also suggested that isoform switching associated with fat and glucose metabolism contributes to human fatty liver disease [6, 15], the related mechanisms in chickens remain unclear, limiting the application of the comparative knowledge between these two species.

The descriptive values from DGE analysis cannot cover the dynamic expression of multiple transcript isoforms of these genes [16]. As DGE analysis solely detects changes in the total gene expression level (the sum of all transcript isoforms), identifying variations in the expression levels of specific transcript isoforms are limited. Resultantly, it fails to provide detailed information about the transcript isoforms that are expressed differentially [12]. Moreover, the differential expression of one or more transcript isoforms does not necessarily cause the differential expression of the entire gene [12, 16]. Therefore, it is not necessary to identify genes with altered expression of their transcript isoforms as differentially expressed genes [12].

Changes in the transcript isoforms can only be examined using transcript-level analysis – also known as differential transcript usage (DTU) [12]. Along with DGE analysis, DTU analysis can uncover the intricate changes in the expression of target transcript isoforms associated with the diseases, enabling improvements in treatment and prognosis [12, 16]. Differential transcript usage analysis can potentially help unravel the mechanisms underlying fatty liver disease that DEG analysis alone failed to resolve. With further cross-species comparison between chickens and humans, we can also obtain novel transcript isoforms with biological as universal markers for this disease in both species.

In this study, we conducted DGE and DTU analyses on liver samples from humans and chickens with fatty liver disease. Our goal was to identify the orthologous genes that exhibited alterations in gene expression and transcript isoform usage across species. Using this approach, we successfully identified candidate genes that met these criteria, deepening our understanding of these diseases and potentially leading to the identification of novel cross-species biomarkers in the future.

## Materials and Methods

### Ethical approval

This study purely involves the use of public datasets and falls into the study category that does not require ethical review by our university committee (RMUTTO Institute for Research and Development committee).

### Study period and location

The study was conducted from October 2022 to February 2023 at the Department of Veterinary Science, Faculty of Veterinary Medicine, Rajamangala University of Technology, Tawan-OK, Chonburi, Thailand.

### Sample datasets

We obtained the RNA sequencing datasets of chicken and human liver samples from the sequence read archive database (https://www.ncbi.nlm.nih.gov/sra) (Table-1) (Accession numbers GSE185051 [17] and GSE111909 [9] for humans and chickens, respectively). In detail, the chicken datasets were derived from biopsies of the first generation of Jingxing–Huang chickens (three birds with a fatty liver condition and three control birds) [9]. For human datasets, clinically indicated ultrasound-guided liver biopsies were collected from NAFLD patients (n = 15) and healthy candidates (n = 5) [17].

**Table-1:** Chicken and human datasets.

Dataset	Species	Condition	Description
SRR6844895	Chicken	Normal	Liver biopsy of normal chicken
SRR6844896	Chicken	Normal	Liver biopsy of normal chicken
SRR6844897	Chicken	Normal	Liver biopsy of normal chicken
SRR6844898	Chicken	Fatty	Liver biopsy of chicken with fatty liver disease
SRR6844899	Chicken	Fatty	Liver biopsy of chicken with fatty liver disease
SRR6844900	Chicken	Fatty	Liver biopsy of chicken with fatty liver disease
SRR16126212	Human	Normal	Liver biopsy of normal candidate
SRR16126210	Human	Normal	Liver biopsy of normal candidate
SRR16126211	Human	Normal	Liver biopsy of normal candidate
SRR16126213	Human	Normal	Liver biopsy of normal candidate
SRR16126209	Human	Normal	Liver biopsy of normal candidate
SRR16126159	Human	Fatty	Liver biopsy of NAFLD patient
SRR16126184	Human	Fatty	Liver biopsy of NAFLD patient
SRR16126186	Human	Fatty	Liver biopsy of NAFLD patient
SRR16126188	Human	Fatty	Liver biopsy of NAFLD patient
SRR16126192	Human	Fatty	Liver biopsy of NAFLD patient
SRR16126194	Human	Fatty	Liver biopsy of NAFLD patient
SRR16126196	Human	Fatty	Liver biopsy of NAFLD patient
SRR16126160	Human	Fatty	Liver biopsy of NAFLD patient
SRR16126162	Human	Fatty	Liver biopsy of NAFLD patient
SRR16126168	Human	Fatty	Liver biopsy of NAFLD patient
SRR16126172	Human	Fatty	Liver biopsy of NAFLD patient
SRR16126180	Human	Fatty	Liver biopsy of NAFLD patient
SRR16126185	Human	Fatty	Liver biopsy of NAFLD patient
SRR16126187	Human	Fatty	Liver biopsy of NAFLD patient
SRR16126189	Human	Fatty	Liver biopsy of NAFLD patient

DTU=Differential transcript usage, NAFLD=Non-alcoholic fatty liver disease

### Experimental design

**Figure-1:**
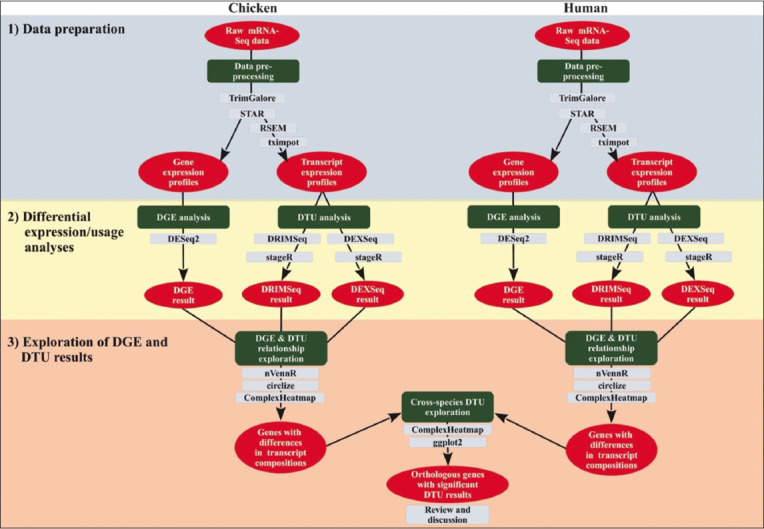
Experimental workflow. The workflow consisted of three sections, as follows: (1) Data preparation, (2) differential expression/usage analyses, and (3) exploration of differential gene expression and differential transcript usage results. The red eclipses indicated the input/output data types used in each analytical process represented in the rounded green squares. The gray, rounded squares indicated the software or package utilized in each analytical process.

The experiment consisted of three sections: (1) Data pre-processing; (2) DGE and DTU analyses; and (3) evaluation of the DGE and DTU results (Figure-1). We pre-processed human and chicken raw messenger RNA-Seq datasets in the “Data pre-processing” section. For DGE and DTU analyses, we employed these pre-processed datasets to identify the differentially expressed genes and transcripts. Finally, we explored the relationships between DGE and DTU results and identified the human-chicken orthologous genes with significant DTU results in the “Evaluation of DGE and DTU results” section.

### Data preparation

#### Data pre-processing

We pre-processed the liver sample datasets based on a previously described procedure with slight modifications [18, 19]. Specifically, we utilized the “TrimGalore” program (https://github.com/FelixKrueger/TrimGalore) for sequence trimming, which removed any contaminated adapter and disqualified sequences that were <110 nucleotides long with a mean Phred score below 30. We used the “STAR” software (https://github.com/alexdobin/STAR) to align the passed sequences to their corresponding genomes: GRCg7b and GRCh38 for the chicken and human data, respectively. During the alignment process, the software counted the number of reads per gene for all datasets. The transcript isoform expressions were quantified from the aligned results using the “RSEM” software (https://github.com/deweylab/RSEM). We then imported and transformed the results as Transcripts per Million values using the “tximport” package [20].

### Differential expression/usage analyses

#### Differential gene expression analysis

We conducted DGE analysis using both the read count data of chickens and humans using the “DESeq2” package with batch correction through the “svaseq” method (false discovery rate [FDR] ≤0.001 and log2-fold-change ≥2) [19]. In this study, we referred to the significant differentially expressed genes obtained from the DGE analysis as “DGE genes.” To obtain the batch-corrected gene expression matrices for both chicken and human data, we applied the “removeBatchEffect” function of the “limma” package [18, 19].

#### Differential transcript usage analysis

We employed the “DRIMSeq” and the “DEXSeq” packages for DTU analysis, following the guidelines [12]. After filtering genes with only one transcript isoform and the genes with a very low abundance of transcripts, we applied the test of DTU between normal and fatty liver samples using both “DRIMSeq” and “DEXSeq” models. We utilized the “stageR” package to screen and confirm the significant genes obtained from the DTU analysis (FDR ≤0.05 with SD per sample proportion ≥0.1). In this study, we referred to the genes with significant differences in their isoform transcript usage, based on the DTU analysis, as “DTU genes.”

## Evaluation of DGE and DTU results

### Relationship between DGE and DTU relationship

We examined the connection between DGE genes and DTU genes. We listed and illustrated the interactions between these gene sets using union, intersect, and complement in a Venn diagram created by the “nVennR” package. To show the expression levels of all interacted genes, we utilized circular heatmaps created by the “circlize” and “ComplexHeatmap” packages.

### Exploring cross-species DTUs

We evaluated all the orthologous DTU genes from both chicken and human DTU analyses [21] using the “ComplexHeatmap” package to illustrate their expression patterns across both species. As this study focuses on the genes with homogeneous expression patterns in normal and fatty liver sample groups across the human and chicken datasets, we only targeted these genes and created expression level bar graphs for each transcript isoform using the “ggplot2” package. We have summarized all transcript isoforms with the same expression trend as their respective genes (Kruskal–Wallis-test, p ≤ 0.05). Furthermore, we reviewed and discussed the possible roles of these selected DTU genes in fatty disease development or fat metabolism.

## Results

### Exploring the interaction between DGE and DTU genes in fatty liver samples from both humans and chickens

**Figure-2:**
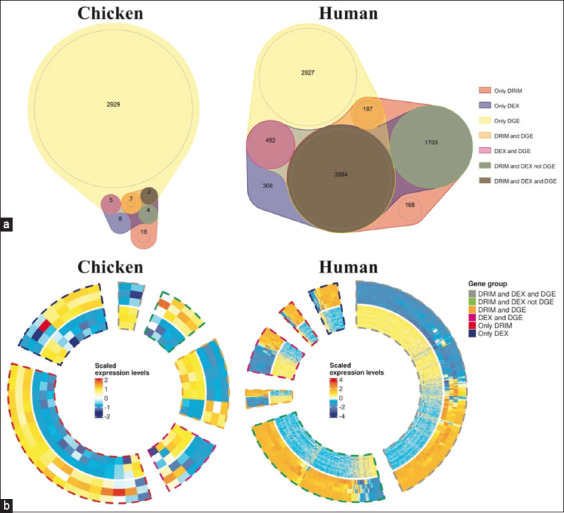
Interaction between differential gene expression (DGE) and differential transcript usage (DTU) genes. The figure illustrated the interaction between DGE and DTU genes from chicken and human analyses. The Venn diagrams showed the number of genes in each label category (a), and the circular heatmaps demonstrated the expression patterns of the interacted genes (b). The label’s description was as follows: (1) “only DRIM” indicated significant DTU genes uniquely obtained from the DRIMSeq model with no difference in their overall gene expressions. (2) “only DEX” indicated significant DTU genes uniquely obtained from the DEXSeq model with no difference in their overall gene expressions. (3) “only DGE” indicated DGE genes with no difference in their transcript isoform usage. (4) “DRIM and DGE: Indicated significant DTU genes uniquely obtained from the DRIMSeq model and significantly different in their overall gene expressions. (5) “DEX and DGE” indicated significant DTU genes uniquely obtained from the DRIMSeq model and significantly different in the overall gene expressions. (6) “DRIM and DEX not DGE” indicated DTU genes significant in both DRIMSeq and DEXSeq models but not different in their overall gene expressions. (7) “DRIM and DEX not DGE” indicated DTU genes significant in both DRIMSeq and DEXSeq models and also different in their overall gene expressions.

The Venn diagrams in Figure-2a demonstrate the interaction between DGE and DTU genes acquired from analyses of both chicken and human samples. The plots also separated the results obtained from the “DRIMSeq” and “DEXSeq” models for DTU genes (labeled as DRIM and DEX in Figure-2a, respectively). While the number of DGE genes with no difference in transcript isoform usage obtained from both species were comparable (“only DGE” in Figure-2a), the DTU results were remarkably different in both gene numbers and proportions (all labels other than “only DGE” in Figure-2a).

To further investigate the expression patterns of chicken and human DTU genes across normal and fatty liver samples, we used circular heatmaps (Figure-2b). The hierarchical clustering revealed homogeneous expression patterns of most DTU genes among normal and fatty liver samples (outer and inner layers of expression rings in Figure-2b, respectively).

### Identification of DTU genes exhibiting consistent cross-species expression patterns in fatty liver disease

**Figure-3:**
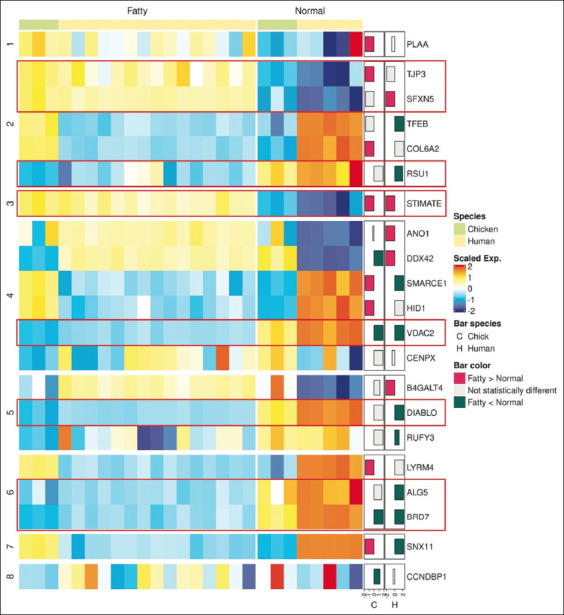
Expression pattern of cross-species differential transcript usage (DTU) genes. The heatmap depicted expression patterns of DTU genes between fatty (Fatty) and normal (Normal) conditions across species (Chicken and Human). The left-side bar plots demonstrated the mean expression differences between fatty and normal liver samples in both chicken (C) and human (H). The significant differentially expressed genes were plotted in pink or green bar colors, as described in the legend. Based on their comparable cross-species expression patterns, we selected seven candidate DTU genes: ALG5, BRD7, DIABLO, RSU1, SFXN5, STIMATE, TJP3, and VDAC2 (highlighted in red rectangles).

To illustrate the expression patterns of orthologous DTU genes in chickens and humans, we created a heatmap to highlight the similarities and differences across both species (Figure-3). Based on the similar cross-species expression patterns (red rectangles in Figure-3), we selected seven DTU genes - *ALG5*, *BRD7*, *DIABLO*, *RSU1*, *SFXN5*, *STIMATE*, *TJP3*, and *VDAC2 -* for further analyzing their functions.

*ALG5* encodes glycosyltransferase, a pivotal enzyme in liver fat metabolism [22–24]. The protein encoded by *BRD7* acts as a coactivator/corepressor in various cellular processes and has been implicated in protection against glucose intolerance and insulin resistance, an important contributing factor to fatty liver development [25]. *DIABLO* can promote liver cell apoptosis in liver disorders [26, 27], including in NADH progression [26]. *RSU1* encodes a Ras signaling negative regulator related to fat accumulation [28, 29] and insulin resistance [11, 30]. *SFXN5* encodes a mitochondrial transmembrane protein participating in lipid membrane trafficking [31–33] with its upregulation evidence in the fatty liver condition [31]. *STIMATE* encodes a store-operated Ca^2+^ junction regulator that indirectly interacts with the STIM1 calcium channel regulatory protein, whose dysfunction is related to fatty liver disease [34]. *TJP3* encodes a tight junction transmembrane protein that controls lipid diffusion and its slight dysfunction has been implicated in fatty liver disease [35]. *VDAC2* encodes for a protein that directly contributes to fatty liver development by regulating mitochondrial glucose and facilitating fatty acid oxidation [36–38]. For further discussion, we summarized the possible roles of all candidate DTU genes in Table-2 [22–29, 31–38].

**Table-2:** Summarized possible roles of proteins encoded by candidate DTU genes.

Gene	Possible role of protein encoded by the gene	References
ALG5	Induce protein N-linked glycosylation of various proteins controlling fat metabolism	[22-24]
BRD7	Act as both coactivator and corepressor of various genes regulating glucose intolerance related to development of fatty liver disease	[25]
DIABLO	Integrate its apoptotic promoting function in the liver cells during disease development	[26, 27]
RSU1	Act as a negative regulator of Ras signaling which promotes the liver’s fat accumulation and insulin resistance	[28, 29]
SFXN5	Possibly have role in lipid’s membrane trafficking	[31-33]
STIMATE	Act as a regulator and coregulator of calcium channel which might link with Ca^2+^metabolic dysfunction in fatty liver	[34]
TJP3	Might be a tight junction transmembrane protein controlling passage of lipids movement of lipids into or out of the liver	[35]
VDAC2	Implicate its role in the of glucose uptake regulation and fatty acid oxidation of the mitochondria	[36-38]

DTU=Differential transcript usage

**Figure-4:**
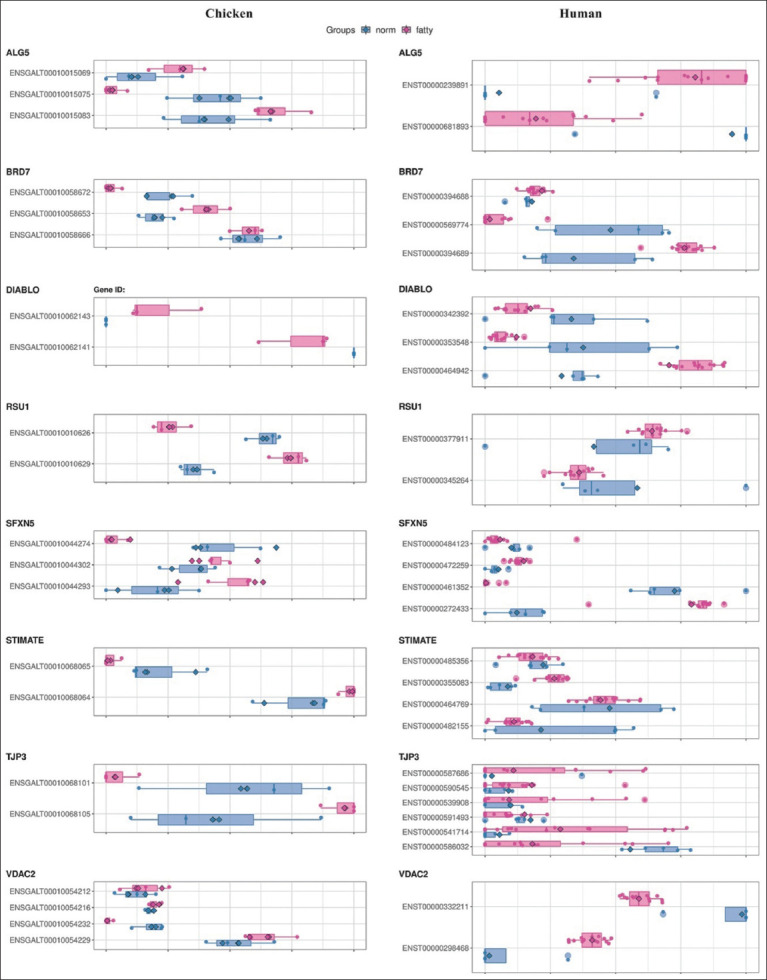
Expression levels of transcript isoforms for candidate differential transcript usage genes. The blue and pink bars depicted the expression levels of normal and fatty liver samples, respectively. We identified all transcript isoforms using Ensembl ID, with “ENSGALT” and “ENST” as the identifiers for chicken and human transcript ID, respectively. The transcript isoforms that exhibited statistical differences are in Table-3 (Kruskal–Wallis-test, p = 0.05).

To understand which transcript isoforms were responsible for the gene expression patterns observed in normal or fatty liver samples, we examined the expression levels of all transcript isoforms of candidate DTU genes using bar graphs (blue and pink bars in Figure-4). Table-3 summarizes all transcript isoforms with the same expression trend as their respective genes in diseased liver samples (Kruskal-Wallis-test, p ≤ 0.05). These selected transcript isoforms were most likely responsible for the observed differential expression patterns.

**Table-3:** Summary of the significant transcript isoforms with same trend of their genes in fatty liver samples.

Common gene name	Transcript isoforms		Expression levels compared to normal liver samples

	Chicken	Human	
ALG5	ENSGALT00010015075	ENST00000681893	Lower
BRD7	ENSGALT00010058672	ENST00000569774	Lower
DIABLO	ENSGALT00010062141	ENST00000353548	Lower
RSU1	ENSGALT00010010626	ENST00000345264	Lower
SFXN5	ENSGALT00010044293	ENST00000272433	Higher
STIMATE	ENSGALT00010068064	ENST00000355083	Higher
TJP3	ENSGALT00010068105	ENST00000541714	Higher
VDAC2	ENSGALT00010054232	ENST00000332211	Lower

## Discussion

In this study, we successfully identified significant transcript isoforms in both chicken and human liver samples with fatty liver disease among the candidate genes. Since these candidate transcript isoforms might participate in fat metabolism, they could provide better insight into the pathogenesis of fatty liver and even become novel cross-species prognostic and therapeutic biological transcript markers in the future. Although the potential outcomes of the study were promising, there are some limitations regarding the data samples. The primary concern was the limited availability of chicken datasets, which could contribute to a restricted number of chicken DTU genes (Figure-2). In addition, the severity of fatty liver samples between chickens and humans could not be compared as no practical guidelines are available for such cross-species comparison. These factors might reduce the sensitivity of the cross-species analyses by reducing the number of candidate genes obtained.

In addition to these limitations, the scarcity of chicken DTU genes acquired from this study also limited the biological reasoning. The Ensembl database obtained from the “BioMart” package [39] revealed that the number of identified genes in chickens was only about half of that in humans. Furthermore, the approximate ratio of transcript isoform numbers per gene in chickens was almost half that in humans. Therefore, we should anticipate a lower number of DTU genes in chickens when compared to humans. Due to this limitation, our study included all the chicken DTU genes obtained from “DRIMSeq” and “DEXSeq” models for subsequent cross-species analysis.

The previous studies have indirectly suggested the existence of cross-species DTU genes between mice and humans [6, 15]. However, this study was the first to directly identify DTU genes shared between chickens and humans concerning fatty liver disease. Since the candidate DTU genes should exhibit similar expression patterns to perform similar functions across species, we selected seven DTU genes with matching expression patterns in humans and chickens (Figure-3). Consistent with our expectations, the previous research has linked these candidate genes to cellular mechanisms involved in fat and glucose metabolism that could potentially contribute to the development of fatty liver disease (Table-2). The results also indicated varying degrees of evidence regarding the involvement of candidate DTU genes in fatty liver disease development, highlighting their differing levels of importance in disease progression. Notably, the changes in the transcript isoforms of specific genes, such as *ALG5*, *BRD7*, and *VDAC2*, which were more likely to impact the progression of the disease due to their direct roles in regulating fat and glucose metabolism. This emphasizes the potential significance of these genes for future studies aimed at verifying their functional roles.

Regarding the encoded protein’s attributes, the differences in the exon numbers in each transcript isoform of the candidate DTU genes can impact their enzymatic activity, localization, and stability. Therefore, the effect of most candidate transcript isoforms identified in this study was unpredictable (Table-3). Although direct evidence indicating the unique functions of these transcript isoforms in fatty liver disease is limited, some studies related to the transcripts indirectly suggested their possible disease-related roles. For instance, the chicken ENSGALT00010010626 and human ENST00000345264 transcript isoforms of *RSU1* might have distinct functions in Ras protein activity because of their additional exon expressions [40]. When downregulated in fatty liver conditions, these isoforms could contribute to the disease’s characteristics.

Besides applying the previously mentioned transcript isoforms as novel diagnostic markers for fatty liver disease, we also wanted to understand the co-occurrence of abnormal transcript regulation in these orthologous genes between humans and chickens. However, further research is required to uncover these shared regulatory mechanisms to understand how the disease affects transcript isoform alteration. This information might provide valuable clues for improving both disease diagnosis and treatment.

## Conclusion

Our study identified novel cross-species DTU genes in fatty liver disease in humans and chickens. The previous studies on these genes supported the idea that these DTU genes most likely acted synergistically in the development and progression of this disease. Future research should focus on verifying the transcript isoform functions and regulations of the candidate DTU genes in fatty liver disease. Despite their cryptic, unique roles, due to their distinct expression patterns in normal and diseased conditions, these transcript isoforms could become potential diagnostic markers for detecting fatty liver disease in the future.

## Authors’ Contributions

KC and TS: Planned the study design, analyzed data, and drafted the manuscript. KC: Collected the data. DP and CN: Reviewed the manuscript. KC, DP, and CN: Performed technical coding correction. All authors have read, reviewed, and approved the final manuscript.

## Supplementary information

The raw count files of human and chicken transcript isoform expressions and other data could be made available on individual request. All abbreviations in this manuscript are as follows:

**Table:**

Abbreviation	Full name/definition
ACACA	Acetyl-CoA carboxylase alpha
ALG5	ALG5 Dolichyl-phosphate Beta-glucosyltransferase
APOA4	Apolipoprotein A4
BRD7	Bromodomain containing 7
DGE	Differential gene expression
DGE genes	Significant differentially expressed genes obtained from DGE analysis
DIABLO	Diablo IAP-binding mitochondrial protein
DPP4	Dipeptidyl Peptidase 4
DTU	Differential transcript usage
DTU genes	Genes with significant differential transcript isoform usage obtained from DTU analysis
FADS2	Fatty acid desaturase 2
FASN	Fatty acid synthase
NADH	Nicotinamide adenine dinucleotide
NAFLD	Nonalcoholic fatty liver disease
RSU1	Ras suppressor protein 1
SCD	Stearoyl-CoA desaturase
SFXN5	Sideroflexin 5
STIMATE	STIM Activating enhancer
TG	Triglycerides
TJP1	Tight junction protein 1
TJP2	Tight junction protein 2
TJP3	Tight junction protein 3
TPM	Transcripts per million
VDAC2	Voltage dependent anion channel 2

DGE=Differential gene expression, NAFLD=Non-alcoholic fatty liver disease
